# Development of high-yield autofluorescent protein microarrays using hybrid cell-free expression with combined *Escherichia coli *S30 and wheat germ extracts

**DOI:** 10.1186/1477-5956-8-32

**Published:** 2010-06-15

**Authors:** Xristo Zárate, David C Henderson, Keenan C Phillips, April D Lake, David W Galbraith

**Affiliations:** 1 BIO5 Institute, University of Arizona, Tucson, AZ 85721, USA; 2 Department of Plant Sciences, University of Arizona, Tucson, AZ 85721, USA

## Abstract

**Background:**

Protein-based microarray platforms offer considerable promise as high-throughput technologies in proteomics. Particular advantages are provided by self-assembling protein microarrays and much interest centers around analysis of eukaryotic proteins and their molecular interactions. Efficient cell-free protein synthesis is paramount for the production of self-assembling protein microarrays, requiring optimal transcription, translation, and protein folding. The *Escherichia coli *S30 extract demonstrates high translation rates but lacks the protein-folding efficiency of its eukaryotic counterparts derived from rabbit reticulocyte and wheat germ extract. In comparison to *E. coli*, eukaryotic extracts, on the other hand, exhibit slower translation rates and poor overall protein yields. A cell-free expression system that synthesizes folded eukaryotic proteins in considerable yields would optimize *in vitro *translation for protein microarray assembly.

**Results:**

Self-assembling autofluorescent protein microarrays were produced by *in situ *transcription and translation of chimeric proteins containing a C-terminal Green Fluorescent Protein tag. Proteins were immobilized as array elements using an anti-GFP monoclonal antibody. The amounts of correctly-folded chimeric proteins were quantified by measuring the fluorescence intensity from each array element. During cell-free expression, very little or no fluorescence was observed from GFP-tagged multidomain eukaryotic plant proteins when *in vitro *translation was performed with *E. coli *S30 extract. Improvement was seen using wheat germ extract, but fluorescence intensities were still low because of poor protein yields. A hybrid *in vitro *translation system, combining S30 and wheat germ extracts, produced high levels of correctly-folded proteins for most of the constructs that were tested.

**Conclusion:**

The results are consistent with the hypothesis that the wheat germ extract enhances the protein folding capabilities of the *in vitro *system by providing eukaryotic ribosomes and chaperones and, at the same time, the *E. coli *S30 extract, which includes an ATP regeneration system, translates the polypeptides at high rates. This hybrid cell-free expression system allows the facile production of high-yield protein arrays suitable for downstream assays.

## Background

High-throughput microarray-based methods have had a considerable impact on biology. DNA microarray technologies are a paradigm, allowing thousands of genes to be studied with a single experiment [1,2], and these have found widespread use in the scientific community. Protein microarrays, on the other hand, have received less attention, largely due to technical difficulties associated with their production. In particular, the time and resources required to produce microarrays comprising many different proteins can be overwhelming. Furthermore, the stability of proteins attached on the microarray surface can be compromised over time by inappropriate environmental conditions and their functionality thereby impaired [3]. Despite these difficulties, protein microarrays remain an important biotechnological tool due to their high-throughput capabilities; applications for parallel analysis of protein-DNA and protein-protein interactions are particularly attractive [4-6]. Antibody arrays are the most common implementation of protein-based microarray technologies [7], in part because of a recognition of the inherent stability of this class of proteins under a wide range of physical conditions. Their applications range from detection and quantification of specific proteins within complex mixtures to the determination of post-translation modifications such as phosphorylation [8,9].

Self-assembling protein microarrays, based on *in vitro *transcription and translation of DNA templates, are conceptually attractive since they have the potential to obviate problems of functional degradation of array performance associated with microarray storage. One of the earliest platforms developed, the protein *in situ *array (PISA), involved protein immobilization on a tag-binding surface inside the wells of a microtiter plate, using PCR-generated DNA fragments as templates [10]. Other examples include production of peptide and protein arrays based on capture of nascent polypeptides [11], and the development of protein arrays 'printed' from DNA arrays [12]. One of the most promising approaches for high-density protein array construction, termed the nucleic acid-programmable protein array (NAPPA), is based on *in situ *transcription and translation of epitope-tagged proteins from DNA elements printed on glass substrates along with an anti-epitope antibody that captures and immobilizes the newly-synthesized protein [13,14].

Common to these self-assembly platforms is the use of cell-free protein translation. Cell-free expression systems comprise lysates that provide the translational machinery for protein synthesis (ribosomes, accessory enzymes, tRNA), amino acids, and an energy supply [15]. Commercially-available cell-free translation systems are based on extracts from *E. coli*, rabbit reticulocytes, insect cells, or wheat germ embryos; they are available in combined (coupled) formats that also provide the elements necessary for mRNA generation from DNA templates [16-18]. The choice of the cell-free translation system depends on the origin of the proteins of interest and downstream applications envisaged after *in vitro *synthesis. In the case of self-assembled protein arrays, the rabbit reticulocyte system has been the most used; the *E. coli *S30 extract has also been used but to a less extent [10-14,19,20]. The use of eukaryotic translation systems is favored to optimize co-translational folding of multidomain proteins and to permit biologically-relevant post-translational modifications [21]. A drawback to the use of eukaryotic systems is that overall rates of translation are much lower than seen for *E. coli *extracts and this results in much lower protein yields [22]. Under specific conditions, for example, using long-term incubations in a continuous-flow system [23], the wheat germ extract can show protein yields similar to that of the *E. coli *S30 extract, but this approach is not compatible with microarray production. A final technical point regarding protein microarrays is the desirability to incorporate into their production a means for high-throughput read-out, fluorescence-based measurements being the most flexible and convenient [24].

Here we describe a novel methodology for the production of protein microarrays using a hybrid cell-free system for protein translation and folding. It is based on the NAPPA approach described above, modified such that every single protein is produced as a chimera, having the coding sequence for Green Fluorescent Protein (GFP) as the C-terminus. An anti-GFP antibody is then used for capture and immobilization of these fluorescent proteins as microarray elements. The advantages of using GFP-tagged proteins are multiple: there is no need to employ exogenous fluorescent dyes to image and quantify protein expression on the array. Since the fluorescent protein is at the C-terminus of the target proteins, and formation of its chromophore depends on proper folding, it automatically provides an indication of the amount of folded protein at each array element. A further modification involves the use of a hybrid cell-free translation system, combining *Escherichia coli *S30 and wheat germ extracts. This extract mixture produces high amounts of folded protein on the arrays without the need for long periods of incubation, or for impractical continuous exchange of reagents associated with energy supply and phosphate removal. Typical arrays were produced within four hours and validated for a number of different eukaryotic proteins, some having rather complex domain structures. These microarrays should be particularly suited for the discovery of novel protein-protein interactions, characterization of complex protein mixtures, and determination of post-translational modifications. The high yields of protein should also be appropriate for high-throughput mass spectrometry analyses at the level of the array elements [25].

## Methods

### DNA constructs

Mutant S65T of the Green Fluorescent Protein, sGFP [26,27], was amplified with primers 5'-CTGACT**TCCGGA**ATGGTGAGCAAGGGCGAGG-3' (BspEI, forward) and 5'-ACTGA**AGATCT**TTATTCGTGCCATTCGATTTTC-3' (BglII, reverse). The 50 μL PCR reaction consisted of 10 ng of template DNA (pIVEX2.7d-GFP), 40 pmoles of each primer, 2 μL of 10 mM dNTP mix, and 2.5 units of PfuUltra DNA polymerase (Stratagene) in PfuUltra reaction buffer. The thermocycler conditions were 95°C for 2 minutes; 30 cycles of 95°C - 30 seconds, 56°C - 30 seconds, and 72°C - 1 minute; and a final extension at 72°C for 10 minutes. pIVEX2.3 d vector (Roche Applied Science) was linearized with XmaI and BamHI, and GFP was ligated into it after BspEI/BglII digestion. This approach retained most of the pIVEX2.3 d multiple cloning site for further manipulations to produce in-frame C-terminal GFP-tagged proteins. Proteins were cloned into pIVEX2.3d-GFP, most of which employed the 5'-NcoI, 5'-NotI, and 3'-XhoI restriction sites; other sites were used as necessary. PCR reactions employed Herculase Enhanced DNA polymerase (Stratagene), and cDNAs were used as templates. *Arabidopsis *cDNAs were obtained from the RIKEN Bioresource Center, Ibaraki, Japan [28,29], from the Arabidopsis Biological Resource Center (Ohio State University, Columbus, OH) [30], or from individual researchers as acknowledged. All constructs were confirmed by sequencing. Plasmid DNA for cell-free expression was purified using Qiagen Spin mini-prep kits. Linear DNA for programmable array printing was prepared from PCR reactions using the DNA constructs made for each target protein as templates. The forward primer contained an amino modification with a 12-carbon spacer for DNA immobilization on the CodeLink substrates. The PCR-amplified region contained the T7 promoter and terminator, the ribosomal binding site, the target protein-GFP sequences, and some more "spacer DNA," mostly from the upstream sequence. The primers used were: 5'-/5AmMC12/CCTCTGACACATGCAGCTCC -3' (forward) and 5'-CTACTTGGAGCCACTATCGAC -3' (reverse). After the PCR, the reaction was desalted and the DNA precipitated with PEG 8K. The pure linear DNA was stored dry until array printing at -20°C.

Table 1 lists the proteins that were expressed on the microarrays in this study. Most of the proteins are from *Arabidopsis thaliana *and they are identified by their respective names, accession numbers, locus identifications, and sizes. These proteins were selected because they are involved in specific physiological processes of interest to us, including photomorphogenesis and hormone metabolism (auxin and gibberellin). Two proteins were included as positive controls (expressed from plasmid DNA), Maltose Binding Protein (MBP) and Glutathione S-Transferase (GST), classic proteins that are widely used for heterologous protein expression in *E. coli *involving affinity chromatography purification steps [31,32]. Also included on the arrays were the bacterial proteins LovR and PixE, the response regulators of the blue-light photoreceptors LovK and PixD, from *Caulobacter crescentus *and *Synechocystis *sp. PCC 6903 respectively [33,34]. These bacterial systems were chosen because they display light-controlled phosphorylation reactions and were therefore envisaged as suitable for troubleshooting the development of assays for kinase activity and phosphorylation status using the protein microarrays. The protein array elements contain a six amino acid domain, LERAPG, between the N-terminal protein and the C-terminus GFP domain, representing remnants of the cloning restriction sites and the original multiple cloning site.

**Table 1:** Proteins expressed on autofluorescent protein microarrays via hybrid cell-free expression

DNA/Protein	[Swiss-Prot]	Reference	Locus ID	Clone*	Size (aa)	Fluorescence Intensity (a.u.)	**CV**^+^
Ankyrin-repeat	Q9FF09	[59]	At5g07840	U14849	175	57167	13.1
MBP	P0AEX9	[31]			368	53806.6875	14.5
GFP	P42212	[26]			242	48381.375	11.9
IMB1	Q56W05	[60]	At2g34900	pda11561	386	37860.1875	14.8
IAA9	Q38827	[61]	At5g65670	C00016(E)	338	30984.0625	7.8
AXR3	P93830	[62]	At1g04250	pda03374	229	29581.875	8.2
SHY2	Q38822	[63]	At1g04240	C00011(F)	189	28544.3125	9.7
GST	P26624	[32]			224	26364.8125	12.9
PIF3	O80536	[64]	At1g09530		524	21759.5625	17.3
PIL5	Q8GZM7	[65]	At2g20180		407	19228.25	19.3
ELF3	O82804	[66]	At2g25930	U24077	339	16343.8125	9.8
PP2C	Q9LME4	[59]	At1g22280	U19121	281	10684.125	12.5
FYPP3	Q9LHE7	[67]	At3g19980	U21104	303	8129.5	9.5
PixE	P74294	[34]			380	5983.25	10.4
TUB1	Q8GZ16	[68]	At1g75780	pda10291	447	4330.3125	9.9
PKS1	Q9SWI1	[69]	At2g02950	U13787	439	3335.0625	9.9
LovR	Q9ABE4	[33]			152	3312.25	9.4
CRY1	Q43125	[70]	At4g08920	U12079	681	3084.25	8.8
CCA1	Q8S8N5	[71]	At2g46830	C105127	526	2739.6875	4.7
NACA2	Q94JX9	[59]	At3g49470	U15737	217	2218.5625	7.1
NDPK2	O64903	[72]	At5g63310	U19177	231	2209.75	8.7
PIL6	Q84LH8	[73]	At3g59060	U16079	300	2166.125	5.4
GID1L1	Q9MAA7	[74]	At3g05120	U17384	345	2143.1875	6.7
FHY1	Q8S4Q6	[75]	At2g37678		202	2123.8125	3.1
DET2	Q38944	[76]	At2g38050	U09847	262	1980.8125	3.7
EID1	Q8LEA8	[77]	At4g02440	pda12208	336	1882.9375	7
PCL1	Q9SNB4	[78]	At3g46640	U22928	323	1821	5.6
SLY2	Q9LUB6	[79]	At5g48170	S63202	157	1747.3125	7.8
CRY2	Q96524	[80]	At1g04400	U19559	612	1715.1875	14.4
RGL1	Q9C8Y3	[81]	At1g66350	U18422	511	1320.0625	10.5
RGA	Q9SLH3	[82]	At2g01570	U13937	587	1286.75	9
							
**No expression**							
**DNA/Protein**							
GAI	Q9LQT8	[74]	At1g14920	U14047	533	1190.4375	2.8
RGL3	Q9LF53	[83]	At5g17490	U60167	523	1139.1875	1.6
TOC1	Q9LKL2	[84]	At5g61380	U21896	618	1093.5625	3.6
SEC	Q9M8Y0	[85]	At3g04240		977	1091.0625	4.1
SPY	Q96301	[86]	At3g11540		914	1054.75	3.4
No DNA						1027.9375	3.9

*Indicates the cDNA clone used to produce T7 promoter-based DNA constructs for protein expression. Clone names that start with 'pda' are from the RIKEN Bioresource Center, Japan; those starting with U or C were purchased from the Arabidopsis Biological Resource Center, Columbus, OH. If no clone is indicated, the cDNA was obtained from an individual researcher.

^+^CV: coefficient of variation.

### Microarray printing

Monoclonal anti-GFP (mouse) was purchased from Rockland Inc., and was employed without further purification; for printing, the antibody was at a concentration of 1.5 mg/mL in 75 mM sodium phosphate (pH 8.5). Antibody arrays were printed, using a GeneMachines Omnigrid 100 Arrayer, onto CodeLink™ activated slides (SurModics^®^). 600 μm anti-GFP elements were printed in 16-element sub-arrays (4 × 4) with 1,100 μm center-to-center element spacing. 15 sub-arrays (three columns by five rows) were printed on each slide. After printing, slides were incubated inside a humid chamber (saturated NaCl) overnight at room temperature. Residual reactive groups on the substrate were then blocked by incubating the slides in Blocking Buffer (10 mM Tris-HCl, 50 mM ethanolamine, pH 9.0) at room temperature for 45 minutes; slides were rinsed with deionized water thoroughly, centrifuged to dryness and stored inside a desiccator at 4°C until needed. In order to print programmable arrays (linear DNA and antibody on each element), the purified DNA was resuspended directly in antibody-printing buffer solution. Volumes were adjusted to get a final concentration of 200-300 ng/μL of linear DNA. This DNA/antibody solution was used directly to print the arrays with the Omnigrid Arrayer. After printing, programmable arrays were treated the same as the antibody arrays mentioned above.

### Cell-free expression

Antibody slides were blocked by incubation in an excess of StabilGuard Choice (SurModics^®^) for 30 minutes at room temperature. The slides were then rinsed with water and spin-dried, and inserted into ArraySlide 24-well frames (The Gel Company). After assembly, a compression-fit gasket acts to physically separate each sub-array. For some experiments, larger frames with 96 wells holding four slides were employed. All transcription-translation reactions were done within the individual wells of these ArraySlide frames. To achieve this, the volume of the cell-free reactions was adjusted to 60 μL and non-ionic detergent, Nonidet P40 (NP-40, Sigma-Aldrich), was included to allow the solution to evenly spread across the bottom of the well, thereby covering the entire area of the sub-array. The *Escherichia coli *S30 protein reaction consisted of the following amounts of reagents from the RTS 100 *E. coli *HY Kit (Roche Applied Science, now sold by 5 Prime): 12 μL lysate (S30 extract), 10 μL substrate mix, 12 μL of amino acid mixture (except methionine), 1 μL methionine, and 5 μL of reconstitution buffer. Included in the expression mixture were 0.5 μL (5 units) of T7 RNA polymerase (Fermentas Life Sciences), 0.5 μL of 10% NP-40, and 1 μg of expression plasmid DNA (in water), up to a final volume of 60 μL. The reaction was incubated for 3 hours at 24°C and 30 minutes at 8°C inside a PCR thermocycler machine. Wheat germ-based protein expression involved the TNT-coupled wheat germ extract system from Promega Corporation. The reactions comprised 25 μL wheat germ extract, 2 μL reaction buffer, 1 μL T7-WG RNA polymerase, 0.5 μL of amino acid mixture (minus methionine), and 0.5 μL of amino acid mixture (minus leucine). The reaction also included 1 μL (40 units) of recombinant RNasin ribonuclease inhibitor (Promega), 0.5 μL 10% NP-40, and plasmid DNA (1 μg); water was added up to a total volume of 60 μL. Reactions were incubated for 90 minutes at 30°C, followed by 1 hour at 15°C. A TNT-coupled rabbit reticulocyte expression system (optimized for protein expression from linear DNA templates) was tested for programmable arrays. The reaction consisted of 40 μL master mix, 1 μL methionine, 0.5 μL 10% NP-40 and 8.5 μL of water; the reaction was incubated at the same temperature and for the same time as the wheat germ. The hybrid cell-free translation reaction included the following reagents from the Roche kit: 6 μL of *E. coli *S30 extract, 5 μL of substrate mix, 6 μL amino acid mixture (except methionine), 0.5 μL of methionine, and 2.5 μL of reconstitution buffer; from the wheat germ TNT Promega kit: 12.5 μL wheat germ extract, 1 μL reaction buffer, 0.25 μL amino acid mixture (minus methionine), 0.25 μL amino acid mixture (minus leucine), and 0.5 μL T7-WG RNA polymerase; also included were 0.25 μL T7 RNA polymerase (Fermentas), 0.5 μL RNasin, 0.5 μL 10% NP-40, and 14.25 μL of water. 10 μL of circular DNA (1 μg total) was added for a final reaction volume of 60 μL. Reactions with mixed extracts were incubated at 24°C for 3 hrs, 8°C for 30 min, and 15 min at 4°C. For any cell-free expression involving programmable arrays, no circular DNA was added to each reaction and the volume was still adjusted to 60 μL. The cold incubation period after expression was included to enhance protein immobilization, through interaction of the GFP-tagged protein with the capture antibody [13], and to maintain protein integrity and avoid protease degradation (4°C incubation) in case the arrays were not washed immediately after expression. Following the cold treatment, the reaction mixtures were removed from the frame wells and each sub-array-well was rinsed three times with 50 mM HEPES (pH 7.8), at 4°C. Slides were removed from the ArraySlide chamber and washed in excess HEPES buffer 3× for 5 minutes each. After a final rinse with ice-cold water, slides were spin-dried and scanned.

### Microarray scanning and data analysis

Fluorescence signals were detected using a GenePix 4200AL microarray scanner, with excitation at 488 nm and emission detected at 511 nm. Arrays were scanned under the same instrument settings at 10 μm resolution. The fluorescence median values from each array element were extracted and averaged for each sub-array/protein (16 elements).

### Western blot analysis and protein expression monitoring

10 hybrid cell-free protein expression reactions and a negative control (no DNA) were set up inside the wells of a microtiter plate in the same manner as described above. Figure eight details the ten GFP-tagged proteins that were expressed. Fluorescence was measured in one hour intervals with a Fluoroskan II fluorimeter (Titerk Instruments, Huntsville AL); excitation at 485 nm and emission detection at 538 nm. The same proteins were analyzed by western blotting; after expression, 5 μL of the cell-free reaction were diluted up to 20 μL with 10 mM TRIS-HCl buffer (pH 8.0), and mixed with 5 μL of 5× sample buffer. Aliquots (10 μL) were added to each well of 12% gels for SDS-PAGE. Proteins were transferred to PVDF membranes, blocked with non-fat milk, then incubated with peroxidase labeled anti-GFP antibody (Rockland Inc.) overnight at 4°C. The next day, X-ray film was exposed to the membrane treated with a chemiluminescence substrate (SuperSignal West Pico Chemiluminescent Substrate, Thermo Scientific).

## Results

**Figure 1:**
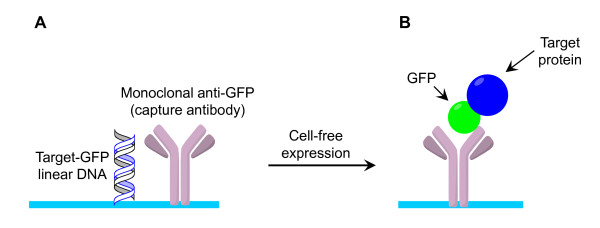
**Production of protein microarrays**. Schematic representation of the production of autofluorescent protein microarrays. **A**. For programmable arrays each element is comprised of monoclonal anti-GFP antibody and linear DNA encoding specific target proteins, robotically printed as multiple sub-arrays. **B**. GFP-tagged proteins are immobilized on array elements after transcription and translation using a cell-free transcription-translation system.

As outlined in Figure 1, the printed array elements contain monoclonal antibody directed against GFP and linear DNA for programmable arrays. Preliminary studies of several commercially-available anti-GFP antibodies allowed identification of a mouse monoclonal antibody having the best capacity to immobilize GFP-tagged proteins on the slide surface (data not shown). In this study, each sub-array, comprising a single chimeric protein, was physically separated with a septum gasket, and because of this, linear DNA was not printed at the locations of the array elements in most of the experiments, since once the microarray slides were blocked and placed inside the frame, the cell-free transcription/translation solution, containing the expression template plasmid, could be added to each well, thereby leading to the production of one protein per sub-array and one sub-array per well. We confirmed the pressure-fit gasket did not permit fluid flow between wells. Over months of experiments using the *E. coli *S30 extract (the Roche RTS kit), considerable variation in expression levels was observed between batches. It was further necessary, once individual kits were opened, to divide the S30 extract into aliquots for storage to maintain activity through avoiding freeze-thaw cycles. In contrast, the wheat germ extract was robust, and did not display batch-dependent variation in activity.

Further preliminary experiments, in which coupled *in vitro *transcription/translation was done using only the S30 *E. coli *extract for translation, defined the basic experimental configuration for detection of immobilized fluorescent proteins, predominantly GFP but also applicable to red fluorescent protein (RFP), using a standard microarray scanner equipped with an additional 488 nm laser (Additional file 1).

**Figure 2:**
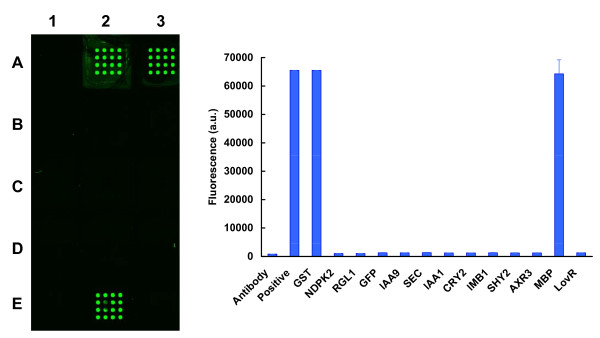
**Analysis of cell-free expression levels obtained using an *E. coli *S30 extract incubated under standard temperature conditions (30°C)**. Microarrays were scanned at 488 nm using a MDS GenePix Autoloader 4200AL, and fluorescence detected using a bandpass filter centered at 511 nm. **Left Panel: **The array surface is divided into three columns and five rows. High levels of fluorescence are observed only for sub-arrays A2, A3, and E2, representing the positive control (purified GST-GFP in the presence of Stabilguard), and the products from GST and MBP. **Right Panel: **The amounts of fluorescence were quantified from the scanned images, and the data presented in the order of sub-arrays A1, A2, A3, B1.....E1, E2, E3.

**Figure 3:**
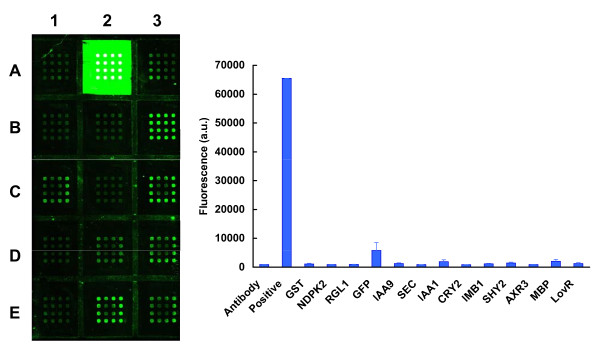
**Analysis of cell-free expression levels obtained using a wheat germ extract**. Microarrays were scanned at 488 nm using the MDS GenePix Autoloader 4200AL. **Left Panel: **Fluorescence is detectable above background for most templates, but high levels of fluorescence are observed only for sub-array A2, representing the positive control (purified GST-GFP in Stabilguard). The microarray was scanned twice, the first time for the purposes of element quantification, and the second time, with the gain settings increased, to allow visualization of low-level expression; this results in saturation of the image in sub-array A2. **Right Panel: **The amounts of fluorescence were quantified from the scanned images, and the data presented in the order of sub-arrays A1, A2, A3, B1.....E1, E2, E3.

Using these conditions, the first set of experiments explored the capacity of the S30 extract to produce recombinant proteins. With transcription/translation being done under standard incubation temperatures (30°C), only two of the recombinant proteins provided fluorescent signals at the array elements when captured by the anti-GFP antibody. These were MBP and GST (Figure 2). In contrast, when incubation temperatures were lowered to 24°C, expression was detected for most of the recombinant proteins, although with great variation in expression levels (Additional file 2). 24°C was therefore employed for all further experiments that included the S30 extract. The next set of experiments explored the corresponding capacity of the wheat germ system (Figure 3). In this case, detectable signals above that of the negative control were observed for all recombinant proteins, but the overall signal intensities were disappointingly low.

**Figure 4:**
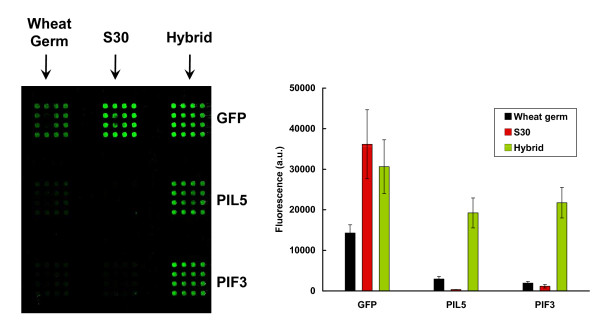
**Comparison of cell-free expression levels obtained using an *E. coli *S30 extract, the wheat germ extract, and a hybrid system that combines the wheat germ and S30 extracts**. Transcription/translation was done of the GFP control, and of the *Arabidopsis *PIL5 and PIF3 proteins. Microarrays were scanned at 488 nm using the MDS GenePix Autoloader 4200AL. **Left Panel: **Fluorescence signals are observed in the scanned image corresponding to cell-free GFP transcription/translation for all three extracts, but are only seen for the two *Arabidopsis *proteins using the hybrid extract. **Right Panel: **Quantification of the relative levels of expression by the three different extracts, for the three proteins. Abbreviations: PIF3, phytochrome-interacting factor 3; PIL5, PIF3-like 5 protein.

**Figure 5:**
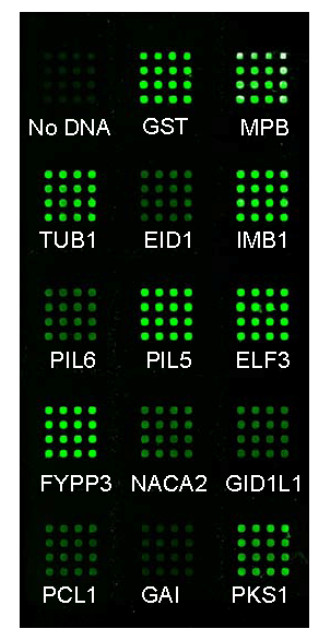
**Production of protein microarrays through *in vitro *transcription/translation using the hybrid cell-free system**. The microarray was imaged using the MDS GenePix Autoloader 4200AL. A total of 14 proteins, including the positive controls MBP and GST, were expressed. In the absence of plasmid DNA, very little fluorescence is seen. Abbreviations: GST, glutathione S-transferase; MBP, maltose binding protein, TUB1, tubulin beta-1; EID1, empfindlicher im dunkelroten licht 1 (increased sensitivity to far-red light 1); IMB1, imbibition-inducible 1; PIL6, PIF3-like 6 (PIF3, phytochrome-interacting factor 3); PIL5, PIF3-like 5; ELF3, early flowering 3; FYPP3, phytochrome-associated protein phosphatase 3; NACA2, nascent polypeptide-associated complex subunit alpha-like protein 2; GID1L1, gibberellin insensitive dwarf 1A; PCL1, phytoclock 1; GAI, gibberellic acid insensitive; PKS1, phytochrome kinase substrate 1.

**Figure 6:**
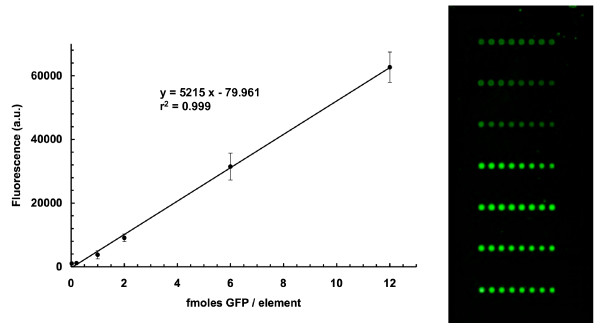
**Calibration curve employed for determination of amounts of protein captured on the microarray surfaces**. Specific amounts of purified GFP protein were printed on CodeLink activated slides. After incubation, fluorescent intensities were determined for each standard to create the calibration curve.

Given that the wheat germ extract expressed a greater variety of proteins, albeit at low yields, and that the *E. coli *extract expressed high yields but only of a few proteins, it was decided to explore the effect of mixing the two extracts, thereby creating a hybrid translation system, and evaluate it for protein expression. A comparison of the relative performances of the *E. coli*, wheat germ, and hybrid translation systems is provided in Figure 4, with the full dataset appearing in Additional file 2. In this case, the amounts produced and captured of two chimeric GFP-protein fusions, corresponding to the *Arabidopsis *proteins, phytochrome-interacting factor 3 (PIF3) and PIF3-like 5 protein (PIL5), were greatly enhanced by use of the hybrid system. This approach was extended to the remaining proteins of this study (Figure 5, and Table 1). In most cases, although the wheat germ extract was able to synthesize some protein, mixing with the *E. coli *extract increased the amount of protein immobilized on each element considerably. Table 1 indicates the fluorescence intensities obtained with the hybrid system for all proteins, which are listed according to level of expression. Proteins exhibiting fluorescence levels lower than that of the negative control plus three times its standard deviation are described as having no expression (based only on the hybrid system), even though the fluorescence intensities of these proteins were in all cases higher than the control. Protein amounts (fmoles/element) were quantified by reference to a standard curve, produced by printing and scanning known amounts of GFP (Figure 6).

**Figure 7:**
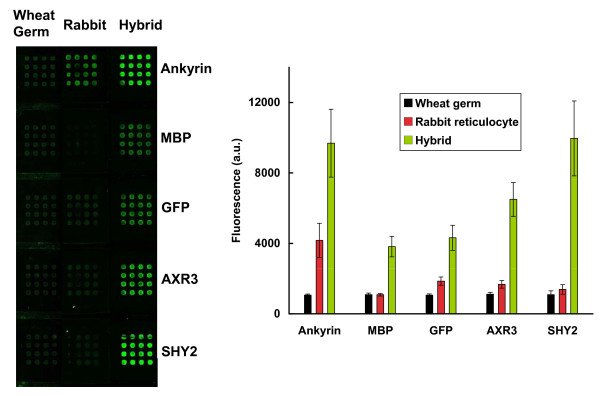
**Comparison of the yields of fluorescent proteins obtained using the wheat germ, rabbit reticulocyte, and the hybrid cell-free expression systems on programmable arrays**. Linear DNA (PCR products) for ankyrin-repeat protein, MBP, GFP, AXR3, and SHY2 was printed with anti-GFP to create programmable arrays. After protein expression with the three different systems, the slide was imaged and the fluorescence quantified.

In the next series of experiments, we evaluated the applicability of the hybrid system for expression of proteins in programmable arrays [13]. Programmable arrays of this type were produced by co-spotting a mixture of linear DNA template and the capture antibody. No plasmid DNA was added to the cell-free transcription/translation mixtures, expression therefore being directed solely by the linear DNA template immobilized on the slide surface. Figure 7 illustrates the fluorescence emission from elements representing five different proteins that were expressed using wheat germ, rabbit reticulocyte (using a kit designed for PCR templates), and the wheat germ/S30 hybrid extracts. In all cases, the highest expression values were obtained using the hybrid extract system.

**Figure 8:**
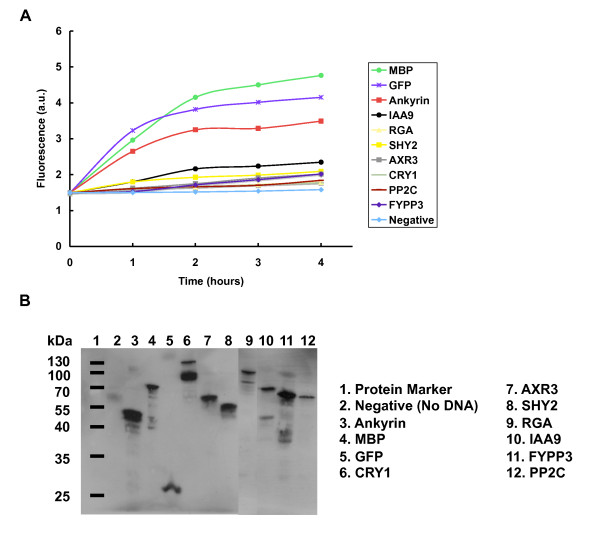
**Characterization of the time courses of expression and final sizes of the recombinant proteins produced by the hybrid extract**. **A**. Time Course. Cell-free expression reactions were set up within the wells of a microtiter plate, and fluorescence emission was quantified each hour over a four hour period using a Fluoroskan II fluorimeter. **B**. Protein Product Characterization. After cell-free expression, the target proteins were analyzed by western blotting, using peroxidase-labeled anti-GFP antibody for detection. Expected sizes in kDa: Ankyrin-repeat, 49.8; MBP, 70.2; GFP 27.3; CRY1, 106.4; AXR3, 55.9; SHY2, 52.1; RGA, 95.3; IAA9, 66.2; FYPP3, 62.6; PP2C, 60.5.

In a final series of experiments, we explored the time-dependence of synthesis of the process of *in vitro *transcription/translation using the hybrid extract, and we verified the sizes of the resultant recombinant proteins using western blotting (Figure 8). All proteins were of the appropriate sizes, although some additional bands were observed in a minority of the lanes.

## Discussion

Self-assembling protein microarrays represent a platform that is conceptually attractive for high-throughput analysis of proteins. The ability to synthesize proteins *de novo *from pre-spotted DNA elements, utilizing combined *in vitro *transcription and translation systems, in particular avoids problems associated with protein denaturation during microarray storage. It also provides extreme flexibility in terms of the choice of protein elements to be immobilized on the arrays.

To be useful, self-assembling protein microarrays require that array elements be produced at high levels, in native states, and at predefined and unique locations on the array surfaces. As originally described [13], NAPPA arrays employed DNA constructs encoding the proteins of interest fused to a C-terminal GST domain. Capture and immobilization of these chimeric species was done via co-spotting a polyclonal anti-GST antibody with the DNA constructs at the array element locations, these mixed macromolecules being immobilized on an aminosilane surface by chemical crosslinking [13,14]. The amounts of immobilized proteins were then determined through addition of a second, horseradish peroxidase (HRP)-conjugated, monoclonal anti-GST antibody coupled to tyramide signal amplification (TSA) [13]. In the TSA system, HRP activity catalytically produces activated dye molecules that in turn react with tyrosine residues of local proteins. Although TSA detection is highly sensitive [35,36], caution is needed to ensure that it does not saturate and thereby provide misleading conclusions concerning the amounts of proteins synthesized. It should be noted that focusing on the epitope identified by the anti-GST antibody provides little direct information about the folding state of the N-terminal protein contained within the chimeras.

To address these issues, we chose to explore use of GFP not only as an epitope for protein array element capture and immobilization, but also as a monitor of protein production and folding. We established that expressing target proteins as N-terminal fusions with GFP allows their immobilization with a capture GFP antibody based on the same principle as employed by NAPPA arrays using GST [13]. Further, for GFP to form its chromophore, it needs first to be folded correctly. It is generally accepted that this protein can be used as a folding reporter when expressed as a C-terminal fusion with other proteins, the proper folding of the GFP domain being related to the correct folding of the N-terminal moiety [37,38]. The use of GFP provides the additional advantage for characterization of these arrays since the fluorescence intensity also indicates the amounts of properly-folded chimeric protein at each element location. This property of autofluorescence obviates the need for indirect labeling with antibodies that are conjugated with fluorescent dyes, such as was done for NAPPA arrays [13]. Evidently, indirect labeling methods may not correlate linearly with the amount of folded protein present. The high concentrations of TSA reactants and unusually long incubation times in the TSA solution employed in that study [13], likely to result in signal saturation, may be the reason for the relatively-uniform levels of expression recorded for very different proteins across the NAPPA arrays. Other reported protein array platforms that use fluorescent proteins did not use intrinsic fluorescence for detection, instead opting for indirect labeling [12,20]. A final advantage of autofluorescent microarrays is that the use of the labeling dyes, required for detection in non-fluorescent protein arrays, can result in background fluorescence; the lower background intrinsic to autofluorescent GFP arrays results in higher quality microarray images [39].

The next question to be addressed was of efficient production of the chimeric proteins *in vitro*. Our results using the *E. coli *S30 extract for protein synthesis indicated, for most of the *Arabidopsis *proteins that we aimed to express as GFP chimeras, levels of fluorescence not much higher than those of the negative control, although a few proteins, such as AXR3, SHY2, and ELF3, consistently showed very high fluorescence levels. In contrast, use of the wheat germ system led to the production of low levels of fluorescence above background for most proteins, but the highest levels seen for any protein were much lower than those seen using the *E. coli *system for the control and auxin proteins. Through performing the wheat germ cell-free batch reaction a second time (after removing the first reaction mixture), we found we could increase the fluorescence signal. This interesting result may be worthy of further study, but the observed increase was at the cost of longer incubation times and of more extract, and was incompatible with high throughput applications of the microarrays.

It is well established that *in vivo *expression of eukaryotic proteins in *E. coli *can be problematic. Many proteins aggregate, as a consequence of misfolding, to form insoluble inclusion bodies; this is particularly evident for large, multidomain proteins [40]. Issues of misfolding have also been reported when using *E. coli *S30 extracts for protein synthesis *in vitro *[21]. It therefore seems probable that an absence of fluorescence using the *E. coli *system reflects inappropriate folding of the chimeric proteins. The results using the wheat germ extract are consistent with a lower capacity, in terms of yield, of this system to synthesize proteins. To cast further light on this question, we decided to mix the *E. coli *and wheat germ extracts. If it were possible to complement the high protein synthetic capacity of the *E. coli *extract with a capacity for correct folding of eukaryotic proteins provided by the wheat germ system, then we would expect to observe high levels of fluorescence for the different chimeric proteins. Remarkably, when the hybrid system was used in this way, most array elements increased fluorescence, including those representing the majority of the *Arabidopsis *proteins that showed low fluorescence values when translated using the *E. coli *extract alone. The levels of expression still varied across different proteins (Table 1), but these levels were higher than those seen following *in vitro *translation in the presence of wheat germ extract alone. For proteins were the S30 system showed higher fluorescence values than the hybrid, e.g. MBP, GST, AXR3, SHY2, and ELF3, can be due to the dilution of the S30 extract with the wheat germ in the hybrid. These proteins were translated and folded successfully with the S30 extract alone and more protein was produced with more of the prokaryotic extract. The hybrid system also performed better than the rabbit reticulocyte system (Figure 7); this image also indicates that our system is compatible with expression from linear DNA molecules immobilized on the array substrate. This implies autofluorescent protein arrays can be produced from fully programmable arrays, similar to the NAPPA system. Analysis of the protein products, using western blotting, confirms appropriate sizes for most of the chimeric proteins. The presence of multiple bands for some proteins, for example FYPP3 and RGA, indicates the quality of synthesis is protein-dependent, and suggests routine quality assurance should be employed in different applications.

Given that the S30 and wheat germ extracts appear to be complementary and act synergistically, the source of this effect can be discussed in more detail. In terms of the prokaryotic system, its main feature is a very high yield under conditions that proteins express and fold successfully [16]. A further feature is its easy genetic manipulation; different *E. coli *strains have been generated for the specific purpose of increasing protein yields during cell-free expression. For example, strain A19 was created with the aim of stabilizing PCR products in S30 extracts for high-throughput protein expression [41]. The KC6 strain was designed for total amino acid stabilization [42]. Energy regeneration, an important aspect of cell-free protein expression, has been the subject of continuous development associated with the S30 extract. For the Roche kit used in these experiments, efficient ATP regeneration comes from the PANOx system [22]; this system regenerates ATP using phosphoenol pyruvate (PEP) and ADP, catalyzed by pyruvate kinase [43]. A further component, oxalic acid, inhibits the reverse conversion of pyruvate to PEP by endogenous PEP synthase [44]. Pyruvate, provided by the pyruvate kinase reaction, reacts with NAD^+ ^and coenzyme A (CoA) to form acetyl phosphate, which regenerates ATP in excess of the ADP that is produced during protein synthesis [43].

In terms of the eukaryotic system, the wheat germ extract was developed with the primary aim of efficient cell-free protein synthesis. This extract, prepared from homogenized wheat embryos, contains all components necessary for translation [45], and ribosome-inactivating proteins such as tritin and other endogenous translation inhibitors, have been removed to improve its stability [46]. Dialysis can be implemented with wheat germ expression reactions to provide a continuous supply of substrates and removal of inhibitory products, and high yields (up to 4 mg of individual proteins) can be obtained, but only after extremely long incubation times (more than 60 hours) [46]. Such long reaction times are impractical for protein microarray production under high-throughput conditions; further, dialysis chambers compatible with the microarray format are not currently available. Clearly, the hybrid system that we have described is an excellent alternative, since it synthesizes folded polypeptides at a high rate in a single batch reaction.

From the point of existing knowledge concerning protein folding, it is not obvious as to why the two *in vitro *systems complement so effectively. Prokaryotic ribosomes, beyond synthesizing polypeptides at rates faster than eukaryotes [40], are also actively involved in protein folding and can effect this process *in vitro *[47,48]. For prokaryotes, protein folding is generally considered as being a post-transcriptional process; in contrast, eukaryotic organisms are believed to employ a different protein-folding mechanism, polypeptides being folded co-translationally [49,50]. Evidence nevertheless exists that wheat germ and rat liver ribosomes are capable of refolding denatured proteins [51]. This activity of wheat germ ribosomes may be responsible for the folding of proteins rapidly synthesized by the *E. coli *system; the wheat germ extract presumably provides chaperones, cofactors, and substrates that assist protein folding [23,52]. Together these represent reasonable hypotheses as to why the hybrid system is particularly effective.

The molecular mechanism(s) underlying the cooperativity in protein production and expression observed between the bacterial and wheat germ systems might involve the following: (a) high-level protein synthesis on bacterial ribosomes, accompanied by co-translational folding, with components for the latter being supplied by the wheat germ extract, (b) stimulation of eukaryotic protein synthesis and of co-translational folding based on eukaryotic factors, by the *E. coli *extract, (c) post-translational folding of proteins synthesized on *E. coli *ribosomes mediated by the eukaryotic extract, or (d) some combination of these factors.

Protein size and domain structure may also influence folding as reflected by the results that we obtained. Eukaryotic cells contain a much greater number of longer proteins than prokaryotes [53] and these proteins contain more domains [50]. The tendency of polypeptide chains to misfold increases significantly as a function of length [54]. It has therefore been proposed that eukaryotic organisms developed a co-translational mechanism to ensure efficient folding, particularly of these larger, more complex proteins [49]. Prokaryotic organisms exclusively use a post-translational folding mechanism, since their polypeptide elongation rates are considerable faster than those of eukaryotes [50]. This is one reason as to why is it difficult to produce large multi-domain eukaryotic proteins in *E. coli*. The overall size of the protein appears to influence successful translation and folding in the hybrid system (Table 1), since the largest proteins (SEC and SPY) did not display high levels of fluorescence. It should be noted that since these proteins both contain glycosyl-transferase domains, which are membrane-associated, the addition of liposomes might improve their synthesis, as recently demonstrated for other membrane proteins [55,56]. The sizes and structure of individual domains also acted as an influence; for example, the DELLA proteins (RGL1, RGA, GAI, and RGL3), which are all poorly expressed and folded, have variable N-terminal domains that contain a unique DELLA motif, but all share the same multi-domain C-terminus [57,58]. Heterologous expression in *E. coli *of these proteins has been reported but only of their N-terminal domains [58]. Therefore it seems likely the C-terminal region is, in this case, recalcitrant to folding. A final reason for low levels of fluorescence might be that those particular proteins can only be synthesized and folded co-translationally by the wheat germ components.

## Conclusion

Cell-free protein expression systems and protein microarrays are important tools in proteomics. Here we demonstrate a novel method to produce autofluorescent protein microarrays using a hybrid cell-free expression system. The folding capacity of eukaryotic ribosomes and chaperones complements the fast translation rates of *E. coli *ribosomes for greatly increased yield of folded proteins. Expression of the proteins on the arrays as fusion proteins with a C-terminal GFP allows direct measurement of protein expression and folding without the need for exogenous fluorescent dyes or signal amplification steps. It is hoped that with further improvements, protein array platforms will fulfill their potential to identify and characterize proteins within complex mixtures in a high throughput manner.

## Competing interests

The authors declare that they have no competing interests.

## Authors' contributions

XZ made the DNA constructs, designed and printed the microarrays, designed and performed the experiments, analyzed and interpreted the data, and wrote the manuscript. DCH participated in array design, and assisted with data interpretation and drafting the manuscript. KCP made DNA constructs, printed microarrays, and helped with experiments. ADL printed microarrays and performed experiments. DWG edited the manuscript, designed the experiments, and proposed the research as Principal Investigator. All authors read and approved the final manuscript.

## Authors' information

XZ is an Assistant Professor at the Autonomous University of Nuevo Leon, School of Chemical Sciences, San Nicolas de los Garza, N.L. 66451, Mexico. DCH currently is a research molecular biologist at the U.S. Department of Agriculture, Agricultural Research Service, Floral and Nursery Plants Research Unit, Beltsville, MD 20705, USA.

## Supplementary Material

Additional file 1 **Detection of expression and immobilization of Fluorescent Proteins on microarrays using a standard slide scanner**. **A**. Expression of GFP. **B**. Expression of RFP. The elements are 100 μm circles and are spaced by 200 μm. Plasmid DNA coding for GFP or RFP (at 250 ng/μL) was co-printed with GFP and RFP antibodies (1.5 mg/mL) respectively on Full Moon protein slides. After S30-based cell-free expression, fluorescence from both proteins was detected at different settings with a microarray scanner. For GFP, excitation was set at 488 nm and emission at 511 nm. For RFP, the scanner settings were the same as for Cy3 (532 nm excitation, 570 nm emission). Click here for file

Additional file 2 **Proteins expressed on autofluorescent protein microarrays via hybrid, S30, and wheat germ cell-free expression systems**. This comprises the entire dataset for all proteins expressed with the three different cell-free systems, and completes the summary in Table 1. The amount of protein (in femtomoles/element) is also indicated, being based on the calibration curve and the highest fluorescence intensity for each protein. For most proteins, this was obtained from the hybrid system but, for a small minority, with the S30 extract alone. Click here for file
