# Supplementary figures and images for: Development of high-yield autofluorescent protein microarrays using hybrid cell-free expression with combined Escherichia coli S30 and wheat germ extracts
Source: Proteome Sci. 2010 Jun 15;8:32. doi: 10.1186/1477-5956-8-32 (PMC2906421; doi:10.1186/1477-5956-8-32)

## Slide 1
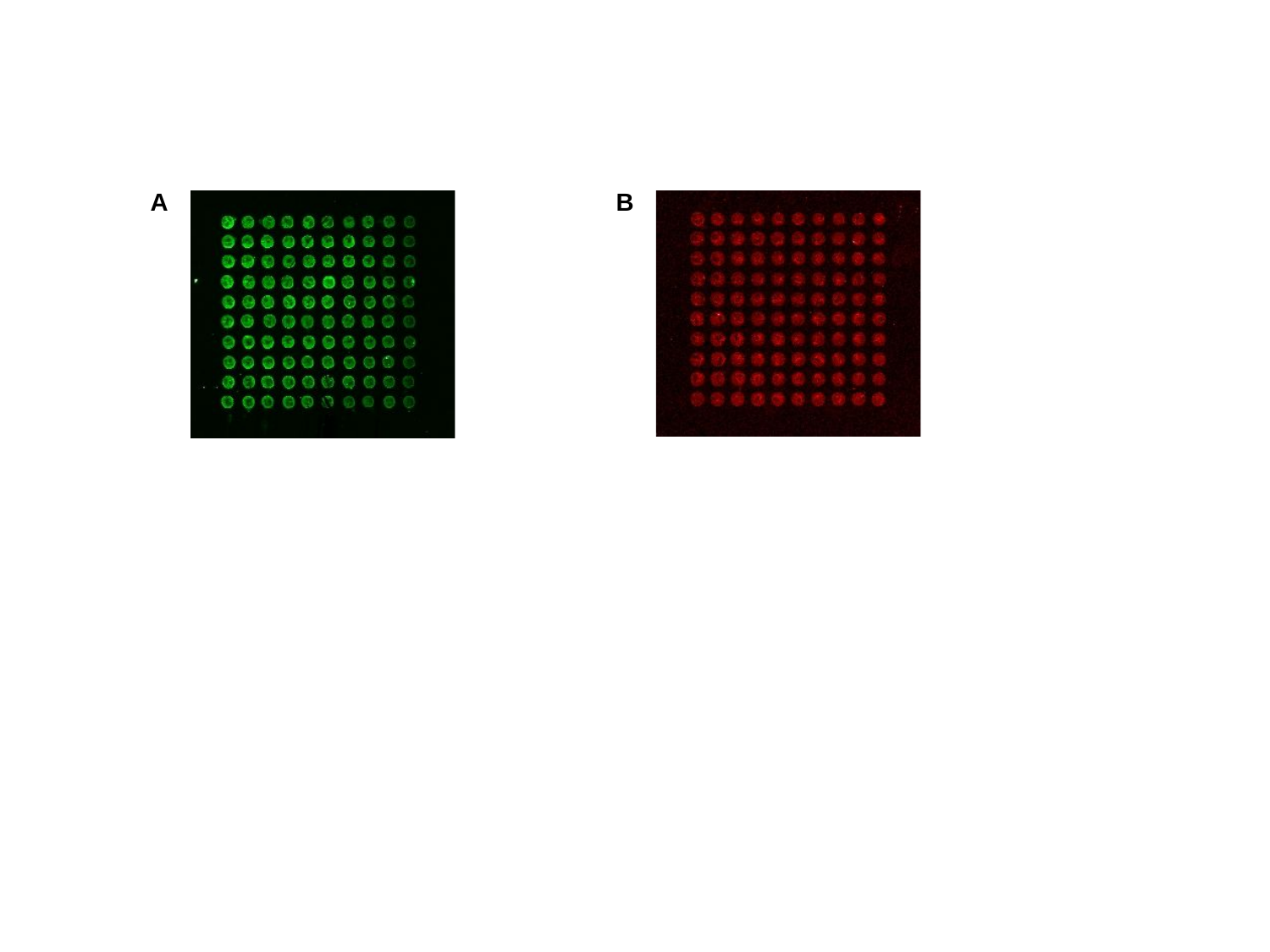

A
B

Supplement: Additional file 1 — Detection of expression and immobilization of Fluorescent Proteins on microarrays using a standard slide scanner. A. Expression of GFP. B. Expression of RFP. The elements are 100 μm circles and are spaced by 200 μm. Plasmid DNA coding for GFP or RFP (at 250 ng/μL) was co-printed with GFP and RFP antibodies (1.5 mg/mL) respectively on Full Moon protein slides. After S30-based cell-free expression, fluorescence from both proteins was detected at different settings with a microarray scanner. For GFP, excitation was set at 488 nm and emission at 511 nm. For RFP, the scanner settings were the same as for Cy3 (532 nm excitation, 570 nm emission). [file 1477-5956-8-32-S1.PPT]
